# The Assessment and Potential Implications of the Myocardial Performance Index Post Exercise in an at Risk Population

**DOI:** 10.4021/cr296w

**Published:** 2014-01-02

**Authors:** Michael Ruisi, Michael Levine, Dennis Finkielstein

**Affiliations:** a Thomas Killip Division of Cardiology Beth Israel Medical Center, New York, NY, USA

**Keywords:** Tei index, Myocardial performance index

## Abstract

**Background:**

The myocardial performance index (MPI) first described by Chuwa Tei in 1995 is a relatively new echocardiographic variable used for assessment of overall cardiac function. Previous studies have demonstrated the MPI to be a sum representation of both left ventricular systolic and diastolic function with prognostic value in patients with coronary artery disease as well as symptomatic heart failure.

**Methods:**

Ninety patients with either established coronary artery disease (CAD) or CAD risk factors underwent routine treadmill exercise stress testing with two-dimensional Doppler echocardiography using the standard Bruce protocol. Both resting and stress MPI values were measured for all 90 of the patients.

**Results:**

Using a normal MPI cut off of ≤ 0.47, the prevalence of an abnormal resting MPI in our 90 subjects was 72/90 or 80% and the prevalence of an abnormal stress MPI in our 90 subjects was 48/90 or 53.33%. The average MPI observed in the resting portion of the stress test for the cohort was: 0.636 with a standard deviation of 0.182. The average MPI in the stress portion of the stress test for the cohort was 0.530 with a standard deviation of 0.250. The P value with the use of a one-tailed dependent T test was calculated to be < 0.05.

**Conclusion:**

We postulate that these findings reflect that the MPI (Tei) index assessed during exercise may be a sensitive indicator of occult coronary disease in an at risk group independent of wall motion assessment.

## Introduction

There are various methods, both invasive and non-invasive, for assessing cardiac function and determining the heart’s performance. Echocardiography remains central in evaluating cardiac structure and function due to its non-invasive nature, high availability and minimal risk profile. The two most commonly used mathematical assessments of left ventricular systolic and diastolic function are ejection fraction and mitral inflow E/A ratio, respectively. Each of these methods has been shown to have considerable limitations. The myocardial performance index (MPI) first described by Chuwa Tei in 1995 is a relatively new echocardiographic variable used for assessment of overall cardiac function. It has been shown in previous studies to be a sum representation of both left ventricular systolic and diastolic function. The MPI therefore represents a measure of global left ventricular function, incorporating both systolic and diastolic variables [1, 2]. It has been proven to be a reliable means of assessing LV function with advantages over the established indices of EF and E/A ratio [1-8]. Furthermore, MPI has been shown to have prognostic value in patients with coronary heart disease as well as symptomatic heart failure [1-13]. Arnlov et al found the MPI at rest to be an independent predictor of cardiovascular mortality in a population sample of elderly men [3]. Mathematically, the MPI is a ratio of time intervals using transmitral diastolic flow and systolic ejection time. The MPI is derived from dividing the sum of the left ventricular isovolumic contraction time (IVCT) and the isovolumic relaxation time (IVRT) by the left ventricular ejection time (ET). The MPI is inversely related to overall LV function, with lower values being more favorable. Traditionally, in previous studies an MPI < 0.47 has been considered within normal range [2]. While there have been numerous reports studying both the prevalence and prognostic value of the MPI in various populations, it has never been evaluated in an at risk cohort of patients during exercise stress testing. The purpose of this study was to observe and assess the MPI during exercise stress in patients with either known coronary artery disease (CAD) or multiple risk factors for CAD (diabetes, high blood pressure, smoking or dyslipidemia).

## Methods

### Study population

The study population included patients with either established CAD or with one or more CAD risk factors (hypertension, dyslipidemia, tobacco use and family history or diabetes mellitus) that were referred for conventional two-dimensional and Doppler exercise stress echocardiography. Patients found to have abnormal resting ejection fraction or resting wall motion abnormalities were excluded from the study. Patients were also excluded if they were in atrial fibrillation, atrial flutter, or demonstrated a paced rhythm. Likewise, patients were excluded if they were unable to give informed consent or failed to understand and participate in the protocol. Patients with poor acoustic windows preventing adequate echocardiographic analysis for the purposes of the study were excluded as well. During the period from January 2010 through September 2010, 318 patients were screened for enrollment in the study. Two hundred and twenty-eight patients were not enrolled due to a lack of traditional risk factors (low risk), presence of arrhythmia, an abnormal resting ejection fraction, or inability to give informed consent. Full demographic data were available for all of the 90 patients who ultimately underwent the protocol (Table 1).

**Table 1:** Demographic Data N=90

Average age (years)	54.3
Sex (male)	38/90 (42.2%)
Coronary artery disease	5/90 (5.5%)
Diabetes mellitus	23/90 (25.5%)
Hypertension	68/90 (75.5%)
Dyslipidemia	55/90 (61.1%)
Tobacco use	17/90 (18.9%)

The presence of six traditional risk factors for CAD (male sex, diabetes mellitus, hypertension, tobacco use, hyperlipidemia and age) as well as the presence of known CAD was recorded for each subject in the study. Two cardiologists performed all exercise stress echocardiograms as well as Doppler analysis and calculations of the MPI indices.

### Stress echocardiography

After informed consent was obtained, routine baseline two-dimensional and Doppler echocardiographic parameters were obtained including simultaneous pulsed wave Doppler through the mitral valve inflow and the aortic valve outflow areas using an apical five-chamber view with the sample volume place between the left ventricular outflow tract and the anterior mitral valve leaflet. If the resting study was normal, a baseline MPI was calculated from these data. The patient then underwent routine treadmill exercise using the standard Bruce protocol with continuous electrocardiographic and blood pressure monitoring. When the patient achieved target heart rate and/or reached the point of fatigue, exercise was terminated and a second set of echocardiographic images was obtained according to the standard ASE protocol. After all 17 wall segments were adequately visualized in the apical view, pulsed wave Doppler through the mitral and aortic valves was reassessed to calculate the post stress MPI. All the echocardiographic images, both resting and stress, were stored digitally to permit later analysis. The MPI was calculated using the following formula:

(*a* - *b*)/*b*=IVCT+IVRT/ET

where *a* is time interval from end to start of transmitral flow, *b* is left ventricular ejection time, IVCT is isovolumetric contraction time, IVRT is isovolumetric relaxation time and ET is ejection time.

### Statistical analysis

The prevalence was calculated using the number of subjects with MPI values greater than the reference value divided by the total in the given subgroup. Data are expressed as mean ± standard deviation. A t test was used for comparisons between the two subgroups. A P value < 0.05 was considered statistically significant.

## Results

During the period from January 2010 through September 2010, 318 patients were screened for enrollment in the study. Of this population, 90 patients were enrolled in the study and underwent the protocolled stress test. Demographic data for these patients are depicted in Table 1, and the relevant echocardiography data for these patients are shown in Table 2.

**Table 2:** Stress Echocardiography Data

Rest portion		Stress portion	
EF ≥ 60%	100%	EF ≥ 60%	100%
Avg rest HR	79.344	Avg stress HR	157.59
Avg A rest (IVCT+ET+IVRT)	438.1	Avg A stress (IVCT+ET+IVRT)	297.10
Avg B rest (ET)	270.167	Avg B rest (ET)	196.83
Avg rest TR velocity	2.448	Avg stress TR velocity	2.914
Avg rest PASP	29.942	Avg stress PASP	43.36

Time: milliseconds (ms); velocity: m/s; pressure: mmHg.

All 90 of the patients were found to have normal wall motion assessment at peak stress indicating a low probability for ischemia. Using a normal MPI cut off of ≤ 0.47 [2], the prevalence of an abnormal resting MPI in our 90 subjects was 72/90 or 80% and the prevalence of an abnormal stress MPI was 48/90 or 53.33% as depicted in Table 3.

**Table 3:** Prevalence and Average MPI at Rest and Stress

Prevalence/Average MPI	Rest	Stress
Prevalence of abnormal MPI in 90 patients	80%	53.3%
Average MPI/standard deviation	0.636 ± 0.182	0.530 ± 0.250

P value < 0.05.

The average MPI observed in the resting portion of the stress test for the cohort was: 0.636 with a standard deviation of 0.182. The average MPI in the stress portion of the stress test for the cohort was 0.530 with a standard deviation of 0.250. The P value with the use of a one-tailed dependent T test was calculated to be < 0.05 (Table 4).

**Table 4:** Paired T test

Variable	Observed	Mean	Standard error	Standard deviation	95% confidence interval
Avg rest MPI	90	0.636	0. 0192	0.182	0.598 - 0.675
Avg stress MPI	90	0.530	0. 0264	0.250	0.477 - 0.583
Difference	90	-0.106	0. 0277	0.2643	0. 051 - 0.161

T = 3.8258; P = 0.0001.

Thirty-two patients of the 90 in the study had an abnormal rest MPI and a normal MPI at stress. Eight of the 90 the patients had a normal MPI at rest, with an abnormal MPI at stress.

Five of our subjects had known CAD with previous percutaneous coronary intervention. Of these five patients: one had a resting MPI index of 0.89 and a stress MPI of 0.462; a second patient had a rest MPI of 0.648, and a stress MPI index of 0.954; the third patient had a resting MPI of 0.78 and stress values of 0.87; the fourth patient had rest MPI of 0.537 and stress of 0.264. The fifth patient with known CAD had both normal stress and resting MPI indices.

## Discussion

The measurement of the MPI is a non-invasive means of assessing global left ventricular function. In a prospective study by Tei et al, the index was found to be more sensitive in the evaluation of diastolic relaxation than the E/A ratio [4]. In other studies the index was found to be an independent prognostic factor for death with more predictive power than ejection fraction or NYHA class [3]. The Tei index has also been shown to have predictive value in relation to the severity of coronary artery disease. Ling et al demonstrated an increase in the Tei index in an ischemic group at peak stress with no significant change in the non-ischemic group during dobutamine stress echo testing [9]. In our cohort of 90 patients with risk factors for CAD or known CAD, all with normal or low probability for CAD on stress echocardiography, the prevalence of an abnormal MPI was less during the stress phase compared to at rest or baseline. Furthermore, the average MPI at stress was less than the average MPI at baseline with statistical significance. Of the five subjects in the cohort of patients with known CAD, two patients had an increase in the MPI at stress, while the other three showed a decrease at stress. While the MPI has been shown to have prognostic value at rest, in our study population its value during exercise stress is unclear. It is possible that a worsening of MPI at stress during peak exercise may be a more sensitive indicator of ischemia than the standard wall motion assessment in this population. For this reason, the MPI may offer additional value as a sensitive screening test for occult coronary artery disease in this patient population. We postulate that an improvement in the MPI in patients at stress with normal resting ejection fractions could possibly indicate the presence of left ventricular contractile and/or lusitropic reserve independent of wall motion analysis. The implications of this are unclear but maybe reflective of a more favorable prognosis in addition to normal wall motion assessment in exercise stress echocardiography. Furthermore, an abnormal MPI at stress could indicate underlying ischemia despite normal wall motion assessment. A study by Norag et al in 2004 demonstrated that the MPI correlated with contractile reserve during low dose dobutamine stress echocardiography in patients with recent myocardial infarction. It increased in patients without reserve and decreased in patients with demonstrated contractile reserve as assessed during low dose dobutamine stress echocardiography. These authors proposed that the change in MPI might be both a quantitative and qualitative assessment of contractile reserve in post recent myocardial infarction patients [14]. More recently in 2009, Stipac et al determined that the presence of contractile reserve assessed by the change in Tei index may identify a favorable prognosis in patients with idiopathic dilated cardiomyopathy [15]. The results in our study suggest that left ventricular MPI improved on average at peak exercise stress in this at risk population. Further studies and analyses are required to fully understand this relationship as well as its potential prognostic significance in exercise stress testing.

Limitations of this study focus on the intra- and inter-observer variability as well as retest variability in the techniques inherent in stress echocardiography. This specifically pertains to the additional Doppler studies of IVCT and IVRT obtained rapidly after the stress phase is completed. Two skilled echo cardiographers performed all of the studies as well as the calculations involved. While only two skilled technicians performed all of the studies, there remains significant potential for variation in overall technique and Doppler measurement from study to study. It is not clear how much this variance would have been reduced with the use of one echocardiographer.

## Conclusion

The MPI or Tei index has been shown to have prognostic implications in both CAD as well as heart failure. However, its significance, prevalence and implications have only been investigated at rest and during low dose dobutamine stress echocardiography. This prospective study investigated the MPI at peak exercise stress in an at risk population. The results from our studied population indicate a decrease in the prevalence of an abnormal MPI at stress compared to rest, as well as a decrease in the average MPI at stress in at risk cohort of patients. We postulate that these findings reflect that with further study, the MPI/Tei index assessed during exercise may be a sensitive indicator of occult coronary disease in an at risk group in addition to or independent of the standard wall motion analysis. Future endeavors are needed to determine the relationship between exercise stress MPI and its prognostic implications.
